# A Letter from Australia

**Published:** 1883-08

**Authors:** I. Field Deek

**Affiliations:** Sidney, N. S. Wales


### A LETTER FROM AUSTRALIA.

DEAR SIR:—I have long thought of writing to you on the subject which I wish to bring before you in this letter. But the loss of two of our principal homœopathic colleagues in this part of the world leads me to delay no longer. You will no doubt hear through the English homœopathic journals in due time, of the death of Dr. Irving, for many years a homœopathic practitioner in Nelson, New Zealand, followed, two or three days afterward, by the death of Dr. Robert Ray, in Melbourne, one of the oldest homœopathic practitioners there. I believe Dr. Irving died from enlargement of the liver. He was the only homœopathic doctor in that part of New Zealand. He enjoyed the confidence of a large clientele and will be greatly missed. Dr. Ray was thrown out of his carriage and died a few hours afterward. He also was greatly respected as a physician. Thus there are two gaps made in our hitherto small and insufficient number of like-minded medical men. I see little hope of our numbers being augumented from England, they need all they have there; but from America, with its homœopathic colleges, and the many graduates who are entering the profession, I think we might expect some help. Homœopathy is extending everywhere. The only want is medical practitioners, and that want is simply everywhere. Here in Sydney, a city of one hundred and sixty thousand, rapidly increasing, we have only four qualified practitioners, and seven unqualified, many of the latter doing well, though very ignorant of medical knowledge generally. There is no one in Queensland, a colony rapidly increasing in prosperity, no one in any inland town in New South Wales. Five of the principal towns in New Zealand, Wellington, Nelson, Napier, Wauganni, Moncayeh, are without homœopathic practitioners. In Dunedin, the principal city, there is only one. I am sure, although I am not as well acquainted with Victoria, that the want is just as universal there. I am writing hastily, but I am sure I have seen enough to show what good openings there are in these parts if we could only get good men to fill them (the climate is delightful and salubrious), but we need good, all-sound men, who are well acquainted (this is my idea) with the best that the dominant school can achieve in both diagnosis and treatment, as well as with homœopathic therapeutics in particular—men who can hold their own in everything. I am sure the status of all could be improved if there were more of us.

Now I have said my say, and I ask you, can you help us in this matter, can you induce any of your colleagues to come and try their fortunes in these transpacific regions? I do not believe they will regret it.

With kind regards, I remain, yours respectfully,

I. Field Deek.

*Sidney, N. S. Wales, May 17th*, 1883.

P. S.—If you can further my object in publishing this, do so. You may remember that I consulted you by letter some years ago about my knee—I received a very kind reply—when I was in Dunedin. I have now been six years in Sydney, and am glad to say my knee is perfectly well.

